# The Developmental Origins of the Social Brain: Empathy, Morality, and Justice

**DOI:** 10.3389/fpsyg.2018.02584

**Published:** 2018-12-14

**Authors:** Chenyi Chen, Róger Marcelo Martínez, Yawei Cheng

**Affiliations:** ^1^Department of Physical Medicine and Rehabilitation, National Yang-Ming University Hospital, Yilan, Taiwan; ^2^Graduate Institute of Injury Prevention and Control, College of Public Health, Taipei Medical University, Taipei, Taiwan; ^3^Research Center of Brain and Consciousness, Shuang Ho Hospital, Taipei Medical University, New Taipei City, Taiwan; ^4^Institute of Humanities in Medicine, Taipei Medical University, Taipei, Taiwan; ^5^Institute of Neuroscience and Brain Research Center, National Yang-Ming University, Taipei, Taiwan; ^6^Department of Education and Research, Taipei City Hospital, Taipei, Taiwan

**Keywords:** empathy, morality, justice, interpersonal harm aversion, inequity aversion

## Abstract

The social brain is the cornerstone that effectively negotiates and navigates complex social environments and relationships. When mature, these social abilities facilitate the interaction and cooperation with others. Empathy, morality, and justice, among others, are all closely intertwined, yet the relationships between them are quite complex. They are fundamental components of our human nature, and shape the landscape of our social lives. The various facets of empathy, including affective arousal/emotional sharing, empathic concern, and perspective taking, have unique contributions as subcomponents of morality. This review helps understand how basic forms of empathy, morality, and justice are substantialized in early ontogeny. It provides valuable information as to gain new insights into the underlying neurobiological precursors of the social brain, enabling future translation toward therapeutic and medical interventions.

## Introduction

The ability to detect contextual cues, such as those of distress and need, has been proposed as the genesis of the mechanism by which basic forms of mimicry and conditioning will naturally evolve into empathy, and later result in helping/moral behaviors (Hoffman, 2007). The perceptual capacity for identifying such cues is significantly meaningful in evolutionary terms, both in regards to the parental care of the offspring, as well as for intra-kin group bonding purposes, as it facilitates group survival. The basic affective circuits on which this ability rests upon emerge much earlier in the brain’s evolution than do higher cognitive capacities (Decety and Svetlova, 2012). The underlying mechanisms that enable mammalian species to discern and react with care to the distress and suffering of another, derive from evolutionarily archaic subcortical circuits (e.g., brainstem, amygdala, hypothalamus, and basal ganglia) and neuro-hormonal systems and processes interrelated with attachment, parental care, and affective sensitivity (Tucker et al., 2005; Decety et al., 2016).

Moral behavior has been theorized to have its roots in this distress detection ability. When human infants perceive distressful cues derived from the other, they themselves will also be affected by this negative emotionality as if it was their own, this due to the children’s premature brain still being unable to effectively differentiate between self and other. Consequently, the infants will try suppressing this distress, but only as a mean to overcome the distress that is produced and mirrored within themselves. This primitive form of egocentric empathy is the precursor for moral behavior. But it is this archaic form of empathy the one that will, across time, and thanks to a future successful differentiation between self and other, turn into genuine, real empathy for the other’s distress (Hoffman, 2007).

Actions to alleviate another infant’s afflictions (e.g., by sharing toys with the other, or comforting him) in emotionally painful situations, are already forms of altruism. As such, these behaviors are pro-social and moral in nature. Altruism at the same time encompasses instrumental helping, whereby an infant acts charitably on behalf of another to help him achieve his goal. But behaviors such as helping and sharing resources inevitably lead to notions such as justice (Warneken and Tomasello, 2009a,b).

The aim of this review is to give account of the neural processes underlying each of the aforementioned aspects of the social brain, beginning with the distress detection ability, going through how this relates to empathy and harm sensitivity, thus, impacting moral behaviors (e.g., inequity aversion), and ending with how the typical processing of others’ distress is affected in different neuropsychiatric disorders, consequently conditioning pro-social behaviors. By the end of this paper, our intention is to have effectively bridged some of the existing psychological and neuroscientific research dealing with the themes of empathy, morality, and justice.

## Perceiving Signals of Distress and Need Across Species

Bird and mammal progenitors who learn to be affected by their offspring’s needs, as a function of their ability to detect such signals, are able to secure the survival of their offspring more successfully than those who remain indifferent due to a lack of such capacity. Consequently, and unsurprisingly enough, a communication system would develop over time and across species where the children’s stylized distress signals would automatically trigger parental care, an ability which later would evolve into even more complex mechanisms such as perspective taking and empathy (de Waal, 2008). Likewise, emotion contagion is an archaic and basic form of hereditary intra-species communication previously observed and researched in many vertebrates (Hatfield et al., 1993). In one study, rats that were taught to action a lever in order to receive food would refrain from doing so if their actions were coupled with the delivery of electrical shocks to an unconcealed, adjacent rat (Church, 1959). Therefore, we can infer that rats perceive a conspecific’s pain as aversive. In another study, rats that were allowed to run free learned to intentionally and quickly open the restrainers of their cage-mates, in order to also liberate them. In another experiment of this same study, when releasing a cage-mate was pitted against obtaining chocolate placed inside a second cage, rats would typically open both of the restrainers, and share the acquired chocolate with said cage-mate (Ben-Ami Bartal et al., 2011). The finding that rats show pro-social behaviors as a result of a conspecific’s distress provides compelling proof for the notion that empathy and helping behavior have their roots in biology. Moreover, the rats’ helping behavior was observed to be reduced by anxiolytic treatment, and associated with sympatholytic corticosterone response (Ben-Ami Bartal et al., 2016). These findings suggest that affect has a fundamental, motivational role in the pro-social behavior of rodents, as shown by the necessity of the rat-helper to resonate with the affective status of the rat-victim.

Nevertheless, there is a downside. Because empathy evolved within the confines of parental-care and cooperatively group coexisting, there’s a great probability that it may still be biased and limited to benefit only “known” or “in-group” members, as members of other groups can be seen as competitors or free-riders (Tomasello and Gonzalez-Cabrera, 2017). For example, it was observed that rats did not help other rats of a different strain, as they were considered strangers. This would change, however, if the freed rat was previously housed with the trapped one. Thus, pair-housing rats would prompt them to help each other, even when they belonged to a different strain (Ben-Ami Bartal et al., 2014). This outcome may provide evidence to support the notion that rats can broaden their pro-social motivational scope to include phenotypically similar others. Consistent with kin selection and reciprocal altruism theory, individuals regarded as familiar, as well as previous cooperators, are favored from an empathy-related standpoint (de Waal, 2008). As stated before, the drawback is the possibility that empathy may not lead all the time and invariably toward morality -as these findings let us observe, and can sometimes be the starting point for wrongful actions by championing exclusively for kin- or self-related interests (Decety and Cowell, 2014b).

## Distress Perception in the Human Infant Brain

At the very early stages of ontogeny in humans, the neonatal brain has been found to have enough sensitivity to discriminate distressful and threat-related voices from emotionally neutral sounds, even when the neonates were in a sleeping state (Cheng et al., 2012b; Zhang et al., 2014).

Lloyd-Fox et al. (2012) demonstrated that 4- to 7-month-old infants already possess the ability to differentiate between voices (e.g., coughing, yawning, throat clearing, laughing, and crying) and non-vocal sounds (e.g., sounds from water running and toys). Moreover, Cheng et al. (2012b) observed that when newborns discern between the emotional voices and the non-vocal sounds, their responses are dominant at the right hemisphere, which is the brain region related to natural speech perception (Alexandrou et al., 2017). In one functional magnetic resonance imaging (fMRI) study, neutral voices (e.g., coughing, sneezing, yawning, and throat clearing), happy voices (laughing), sad voices (crying), and non-vocal sounds (toy and water running sounds) were presented to 3- to 7-month-old babies (Blasi et al., 2011). Infants at this age were found to have activations not only in voice-sensitive regions (the right anterior middle and superior temporal gyri, and medial frontal gyri), but also in brain regions specific to negative voices (i.e., the left orbitofrontal cortex and insula). Since it has been observed that the ability of speech perception, especially for discriminating phonetic contrasts, emerges during the first month of birth (Dehaene-Lambertz and Dehaene, 1994; Cheour et al., 1998; Dehaene-Lambertz and Pena, 2001; Kuhl, 2004), and because of the fact that voice perception emerges earlier than speech perception in human development (Belin et al., 2004; Blasi et al., 2011), it is plausible to conclude that the ability to perceive voices appears before, or at least around birth. This conclusion is supported by previous studies yielding findings that demonstrate that fetal heart rate increases when hearing the maternal voice, and decreases when listening to a stranger’s (Kisilevsky et al., 2003). These results suggest that fetuses already possess vocal identity. In the same manner, Beauchemin et al. (2011) reported that newborns could discern the mother’s voice from a female stranger’s one. Furthermore, according to Cheng et al. (2012b) serial experiments, newborns were sensitive to emotional voices beyond specific language, but it is key to note that the specificity is driven by voice perception *per se*. Thus, it sounds reasonable to infer that newborns exhibit the ability comparable with adults to process the affective information, such as pain and distress, being conveyed through the voice (Schirmer et al., 2005; Fan et al., 2013; Chen et al., 2014, 2015, 2016b, 2017a; Hung and Cheng, 2014). Voices with affective information are presumed to elicit more processing resources than those without affect. Hence, the emergence of the specialization for processing emotions is already advanced at the first days of life.

When visual affective discrimination is involved, studies have shown that between 18 and 24 months of age, and comparable to auditory stimuli, infants show decreased ERPs when exposed to strangers’ faces than when exposed to their mother’s face (Carver et al., 2003). Nevertheless, children’s initial ability to discriminate facial emotions is not as effective, and only significantly improves with age (Boyatzis et al., 1993). Children’s attention to visual cues varies depending on if the stimuli is static or dynamic. The infants’ processing of visual whole-body static images (whether their faced is veiled or not) appears to be immature, but when shown dynamic whole-body stimuli (a more real-life-like scenario), their processing resembles that of adults (Nelson and Mondloch, 2018).

When it comes to the neural underpinnings of visual affective cues processing, a recent review paper by Bachmann et al. (2018) reports that emotional stimuli evokes greater neural responses than neutral stimuli, and that the brain regions observed to have a significant activation during these processes encompass the action observation network (conformed by the inferior frontal gyrus, the premotor cortex, and the inferior parietal lobe), suggesting that human brains interpret others’ actions by employing motor simulations grounded on the motor programs they already possess (Kilner et al., 2007); the mentalizing network (which includes the temporo-parietal junction, the temporal pole, the lateral orbitofrontal cortex, and the dorsomedial prefrontal cortex), making it possible for the individual to infer the other’s mental state (also known as theory of mind) (Frith and Frith, 2006); as well as other regions whose task is to process body motion and form, such as the extra striate body area, the fusiform body area, and the posterior superior temporal sulcus, which interestingly respond selectively to emotional body movements (Downing et al., 2001; Blake and Shiffrar, 2007; Peelen et al., 2007; Pichon et al., 2009; Sinke et al., 2010; Kret et al., 2011; Atkinson et al., 2012). In addition, the amygdala and the hypothalamus have also been observed as having pivotal roles when observing body expressions containing emotional valences, as the former brain region is involved in emotional processing, while the latter holds a crucial role in regards to defensive reactions and in action preparation (Barbas et al., 2003). What’s more, due to self-protection purposes, an appropriate recognition and a suitable response to threatening or dangerous individuals is of utter importance, thus, the processing of this experiences is particularly favored. This is suggested by the fact that emotional content possessing the aversive valences of threat and anger, in contrast to those containing negative valences of fear and sadness, specifically elicit greater activity in brain structures such as the superior temporal sulcus, the premotor cortex, the temporo-parietal junction, the dorsomedial prefrontal cortex, and the lateral orbitofrontal cortex (Pichon et al., 2008, 2009; Peelen et al., 2010).

Bachmann et al. (2018) further posit that when it comes to attentional matters, implicit and explicit tasks appear to activate brain regions differentially, while the hypothalamus, the premotor cortex, and the amygdala are equally activated by both implicit and explicit tasks, other areas, in particular those conforming the mentalizing network, react to explicit judgements of emotionally charged stimuli.

## Affective Arousal/Emotional Sharing

The effective perception of distress will give rise to several other facets involved in human empathy, including affective arousal/emotional sharing, empathic concern, and perspective taking. Each facet of empathy affects moral cognition differently and predicts distinct consequences regarding moral behavior (Decety and Cowell, 2014a). Among them, the affective element comprising empathy evolves earlier than the cognitive aspect. For instance, newborns and infants become significantly distressed immediately after another newborn starts crying (Dondi et al., 1999). What’s more, they themselves also begin crying when this happens, and their cry shows as much distress as that of the infant who initiated it. Noteworthy, is that this cry is far from just a form of imitation, as it is not as intense when they are exposed to a chimpanzee’s cry, nor even when they are exposed to a recording of their own cry (Simner, 1971; Sagi and Hoffman, 1976; Martin and Clark, 1982). Furthermore, human infants (around 10 weeks of age) can readily imitate expressions of fear, sadness, and surprise (Haviland and Lelwica, 1987), fitting individuals for future empathy-based connections by means of affective interactions with others (Decety, 2010). Notwithstanding, when it comes to this form of imitation, a recent longitudinal study put into question the notion that this ability is present since birth (Oostenbroek et al., 2016). It was found that there was no such imitation by neonates, attributing previous findings to be the cross-sectional nature of the research.

When it comes to emotional sharing, this capacity may be partially underpinned by the mirror neuron system (MNS), which -as observed by electroencephalographic (EEG) studies- appears to be already operating in infants with ages as young as 6 months old (Nystrom, 2008). First observed in the 1990’s in non-human primates, the MNS is a system hypothesized to be the motor behind action understanding and imitation (Lepage and Theoret, 2007), hence, having an important role in perspective-taking and empathy (Nystrom, 2008), assumptions which have drawn much debate in the scientific community. While some research have found evidence just partially supporting these hypotheses (Dinstein et al., 2008), others have argued against these claims to the extent that they even doubt the existence of an MNS in humans (Hickok, 2009), although more recent studies have endorsed the initial assumptions (Woodward and Gerson, 2014), as well as supported the notion that the MNS in the human brain is already present since infancy. Furthermore, Meltzoff (2007) considered the MNS as a possible platform for the foundation of social cognition as a whole. He argued that infants effectively identify and match between actions performed by others and the proprioception arising from their own bodily movements. By registering these equivalences between the acts performed by another and those performed by the self, the infant is able to perceive that the others are ‘like me.’ This recognition of others as having similar perceptions and emotions would form the bedrock and starting point for social cognition. Nevertheless, further research is warranted as to elucidate the issues and intricacies, as well as to bridge the diverging literature, surrounding the MNS.

Moreover, in research using EEG/ERP in both children and adults, there is ample evidence showing that passive observation of visual stimuli depicting physical injuries to body parts, triggers an early component (N2, ∼200-ms) and a late-positive potential (LPP, ∼800-ms) (Chen et al., 2012; Cheng et al., 2014; Fan et al., 2014). The early N2 within a time window of 200 to 300-ms was observed to be modulated by attention to emotionally salient stimuli, and reflects affective arousal or emotional sharing; whereas LPP within 500 to 800-ms indexes cognitive reappraisal and emotion regulation (Li and Han, 2010). Adolescents, in comparison to young adults, elicited an earlier N2 in reaction to another’s pain, and a greater LPP in response to neutral stimuli, demonstrating that affective and cognitive empathy is still developing during the adolescent years (Mella et al., 2012).

Throughout early development, children display enhanced N2 to pain during empathic concern engagement. Larger early N2 ERPs were evoked when perceiving painful stimuli versus when perceiving neutral stimuli. Within the slow wave window of LPP, greater differences between painful and neutral images occurred in the empathic concern condition when compared to the perspective-taking condition. Increased pain-evoked N2 responses in the cognitive empathy condition were also associated with empathy regarding parental disposition (Decety et al., 2018). Multilevel analyses, including neurophysiological responses to different empathic conditions and parental empathy levels, is warranted to demonstrate the important differences between the various aspects in children’s empathy. This provides a thought-provoking link between parental dispositions and the children’s neural workings during early development.

## Early Interpersonal Harm Sensitivity

One recent framework proposed the importance of third-party harm aversion as a necessary platform for constructing morality (Decety and Cowell, 2018). When presented with wooden characters pushing a red circle up (i.e., helping) or down (i.e., hindering) a hill, preverbal infants (6- and 10-month-olds) showed a negativity bias by staring significantly longer at, and reaching for the pro-social character, indicating aversion to anti-social behavior (Hamlin et al., 2010; Hamlin, 2014). In another experiment using the same paradigm, when the red circle was attached with “googly eyes” (social condition), 3-month-old infants preferred the character helping the climber uphill, over the character pushing the climber downhill, but they didn’t show any preference when the “googly eyes” were not attached to the circle (inanimate control condition). The 3-month-olds’ preference for the helpers over the hinderers appeared to indicate specific social evaluation (e.g., a perceptual predilection for uphill-helpers over downhill-pushers) (Hamlin et al., 2010). In addition, when researching on sympathy-related behaviors in 10-month-old infants, one study observed infants’ predilection for victims, as opposed to a preference toward aggressors and neutral objects, after watching as a third-party social interactions containing enacted aggression. This indicates that preverbal infants exhibit sympathy-based responses toward others when they are attacked, even when they don’t display any distress (Simner, 1971; Sagi and Hoffman, 1976). Furthermore, research has showed that 3-year-olds tend to avoid helping adults whose intentions are forthright harmful (Vaish et al., 2010), or tend to protest when observing this actions as a third-party (Vaish et al., 2011). Thus, a primitive form of sympathy toward others, emanating from interpersonal harm sensitivity that goes beyond being a simple response to distress as a consequence of emotional contagion, arises earlier during development than previously concluded (Kanakogi et al., 2013; Lee et al., 2015). In line with these findings, Scott and Baillargeon (2017) have pointed out that previous literature has misreported false-belief understanding, which empathy seemingly predicts (Ferguson et al., 2015), as emerging until the 4 years of age, this due to the experimental design taking into consideration traditional tasks, and excluding nontraditional ones (e.g., spontaneous-response and elicited-intervention tasks). When we take into account the latter, infants under 2 years of age have been seen to already exhibit false-belief understanding. Hence, and given the infants’ premature verbal ability and insufficient information-processing resources (Scott and Roby, 2015), the aforementioned third-party moral evaluations can be considered to be driven by intuitive processes that form the understructure of a natural, untaught and unlearned moral core, molded by natural selection in order to facilitate social cohesion and cooperation (Hamlin, 2015).

Recently, research in developmental neuroscience that has taken into consideration parental dispositions has largely confirmed these behavioral findings, and identifies specific neural mechanisms underpinning early socio-moral evaluations and their correspondence to moral preferences. In one study, infants (ages 12–24 months) watched dynamic visual stimuli depicting cartoon characters intentionally executing either pro-social or antisocial acts, while EEG, time-locked ERP, and gaze fixation were recorded. After watching the animation, the experimenters provided a physical version of the helper character and another of the hinderer, as to assess the infants’ reaching preference. This yielded several specific neural outcomes, which include asymmetrical frontal spectral power densities, eye-tracking differences, and time-locked condition differences in the relatively automatic ERPs component (Nc, within 300 to 500-ms time window), at the time social evaluations were occurring. Children’s reaching preference for the pro-social character, as well as parental sensitivity to injustice for others, could be predicted by the early automatic ERPs component, which were sensitive to the perception of pro-social versus antisocial scenarios (Cowell and Decety, 2015a). This differentiation should be understandably basic in essence, being firmly established in primitive resource allocation to relevant stimuli, and approach/withdrawal dispositions. The domain-general systems concerning attention and self-regulation, control early evaluations of social and moral nature, supporting increasing evidence yielded by the neuroscience of morality.

Another neurodevelopmental study that probed into implicit moral evaluations of antisocial and pro-social behaviors in children aged 3–5 years old showed distinct ERP components for early automatic attention (EPN) while they were watching characters enacting helping behaviors, and later cognitive control (LPP) while watching characters enacting harming behaviors. Noteworthy, later (LPP) waveforms, but not early (EPN) ones, predicted the children’s current generosity (Cowell and Decety, 2015b). These findings further underscore that automatic, intuitive/affective, and cognitively controlled processes are required for what appear to be a basic third-party discernment concerning harming and helping behaviors.

## Inequity Aversion

Taking into account helping behaviors, a considerable amount of research suggests that the predecessors of what motivates justice have evolved and developed due to environmental pressures where stabilized group cooperation was needed for survival purposes (Decety and Yoder, 2017). Observational studies about non-human primates’ natural interactions served as the best opportunity to look into these primates’ understanding of fairness and justice. Some primate species living in unique social systems characterized by exceptional social permissiveness and cooperation (which go all the way from sharing food to child care) have evolved inequity aversion, consequently responding negatively when receiving less reward than their social partners (Brosnan, 2013). As such, these species exhibit enhanced pro-social behavior when compared to other primates. Thus far, the extant findings best back the hypothesis that an aversion to inequity arose jointly with cooperative behavior between unrelated individuals (Price and Brosnan, 2012).

In the studies with a violation-of-expectancy paradigm, devised to assess expectations in human infants regarding equal distribution of resources, 15-month-old infants showed increased attention to unfair (unequal) over fair (equal) outcomes (Sommerville et al., 2013). In another developmental study, 19-month-old children expected a distributor to divide resources equally between two similar individuals. This expectation could be attributed to the sensitivity toward social inequity, rather than to low-level sensory processing, given that it was absent when the individuals were replaced with inanimate objects. Furthermore, 21- to 36-month-olds have been observed to already expect equity in distribution when individual efforts resulting in cooperation are taken into consideration. Hence, infants expected that a hard worker and a slacker should not be rewarded equally (Sloane et al., 2012). Nevertheless, and interestingly enough, it has also been observed that during this age window, rewards are shared more equitably when there is cooperation than when there is just parallel-, non-cooperative work involved (Hamann et al., 2011). The sensitivity degree to fairness as a third-party observer in 15-month-old infants, was seen to be associated to whether they shared toys altruistically or selfishly, denoting that moral appraisal and pro-social behavior are highly interconnected since early in development (Schmidt and Sommerville, 2011). These other-regarding inclinations emerge in a parallel and close knitted manner. The origin of a basic sense of fairness and altruism during infancy plays a pivotal role in the development of human-specific types of cooperation.

However, even though research has seemingly led us in a straight line from empathy to distress relief behaviors, toward inequity aversion, and leading to fairness and justice, it is important to underscore that this bridge needs to be treaded carefully. Empathy can inevitably lead to moral, pro-social behaviors such as altruism (de Waal, 2008), and, as stated before, altruism is greatly associated with morality and fairness (Schmidt and Sommerville, 2011). Nevertheless, not all forms of fairness, such as that stemming from collaboration in which both actors reap rewards, is considered as pro-social or altruistic, due to its underlying selfish nature (Warneken and Tomasello, 2009a). Yet, we can still push past this issue. Research was conducted with 21- and 27-month-old infants, where they had to collaborate with an adult toward the achievement of a joint goal. At one moment during the experiment, the adult simulated that he was unwilling or unable to continue with the task. When the latter occurred, the children tried to re-engage their collaborator with the same magnitude on both scenarios: when they needed their help to reach their individual goal, and when they didn’t need of their participation to do so, irrespectively, and in contrast to when the adult was unwilling to complete such task. Therefore, this study demonstrated that infants in the employed age range were not only capable of understanding the other person’s intentions, but were also able to act altruistically toward them (what is referred to as instrumental helping), rather than just using the other as a mere social tool (Warneken et al., 2012). Due to these later findings, we can now go full circle from distress perception and empathy, passing by affective arousal and harm sensitivity, to inequity aversion and fairness, and back again to empathy and empathically led pro-social behavior.

## Atypical Early/Automatic Processing in Neuropsychiatric Disorders

One source of evidence concerning the crucial role of these primitive forms of the social brain, including automatic distressful voice perception, early affective arousal/emotional sharing, and early sensitivity to interpersonal harm during the development, comes from atypical socioemotional processing as a result of neuropsychiatric disorders.

Previous studies using a passive auditory oddball paradigm to investigate the automatic processes in human voice perception, revealed that people with autism spectrum disorder (ASD) manifest general impairments in affective voice discrimination, as well as in low-level acoustic distinction. In addition to decreased amplitudes of mismatch negativity (MMN), which is a reliable index of the neural representations underlying automatic central auditory perception in response to acoustically matched non-vocal sounds, people with ASD failed to discriminate between happy and angry syllables (thus, having an impaired distress detection ability). Weak amplitudes of angry-evoked MMN were associated to severe autistic traits. As a result, examining emotional MMN may offer an opportunity to facilitate an early diagnosis for infants at risk for ASD (Fan and Cheng, 2014). Early-onset conduct disorder (CD), the major childhood predecessor to antisocial personality disorder in adulthood, also showed an atypical fashion in the processing of distressful voices at the pre-attentive level. The presence of increased differences in MMN between fearful and sad voices, and correlations between MMN and impulsive tendencies in youths demonstrating CD symptoms and a history of delinquent behaviors, helps shed light on the neural mechanisms of aggression (Hung et al., 2013). Atypical neurophysiological responses to threatening voices were also evidenced in patients with chronic schizophrenia, suggesting general impairments of voice perception and acoustic discrimination. The emotional salience processing of voices exhibited atypical characteristics at the pre-attentive level, which were related with positive symptoms in schizophrenia. These results may present evidence for bottom-up (i.e., perceptually based) cognitive remediation strategies, and indicates that emotional MMN may be a potential neurophysiological endophenotype for schizophrenia (Chen et al., 2016a).

In one neuroimaging study, when visual scenarios depicting intentional and unintentional harm were presented to CD with callous unemotional traits, a reduced hemodynamic response was found in the insula, a brain region which plays a crucial role in empathy and emotional awareness. Additionally, a weaker activation of the right posterior insula, as induced by perceiving the harm of others, was associated with more CD symptoms and callousness. Similarly, the reactive aggression scores of CD were associated with hyperactivity in the posterior insula and anterior mid-cingulate cortex, indicating heightened emotional response to provocative stimuli (Michalska et al., 2016). Another study applying electroencephalography and event-related potentials (EEG/ERPs) in juvenile psychopaths, appears to echo with the aforementioned findings. Youths with higher callous unemotional traits exhibited atypical neural dynamics in regards to pain empathy processing in the early stages concerning affective arousal, which was coupled with their comparable insensitivity to actual pain. Their ability to understand others’ intentions, however, was not seen to be affected. Such disassociation between affective arousal and emotion understanding might contribute to generating aggressive behaviors in juvenile psychopaths (Cheng et al., 2012a).

Importantly, the link between pain sensitivity and empathic response was uncovered in adolescents with autistic and CD symptoms (Chen et al., 2017b). When compared to typically developing controls, the tactile pressure pain threshold was lower in the autistic group when compared to the conduct group, whose threshold was higher. The autistic group exhibited decreased ratings of unpleasantness and pain intensity to the sufferings of others than did the controls and the conduct groups. In the autistic and conduct groups, pain intensity scores were significantly associated with unpleasantness ratings to others’ pain. Moreover, the color autistic group significantly differed from the controls in the association between pain threshold values and unpleasantness scores. Importantly, these results may cast some light on the relationship between atypical low-level sensory functioning, for example altered pain sensitivity, and high-level processing of empathy.

## Conclusion

This review sheds some light on the primitive forms of the social brain as observed in early ontogeny. To distinguish among the different facets of empathy, including affective arousal/emotional sharing, interpersonal harm sensitivity, and perspective taking, it is critical to avoid illustrating the complex relationship between morality and empathy in a misleading and equivocal way. The presence of affective arousal/emotional sharing, a basic form of empathy, helps in the understanding of such relations more precisely, as well as assists in elucidating the concepts being used in the current literature and, perhaps, also aids in the abandonment of the muddy concept of empathy (Decety and Cowell, 2014a). The interpersonal harm aversion exhibited in early life, shapes the fundamental nature of moral and social cognition, indicating that such motivational value lies in more general processes, rather than in fully distinct, specific neural regions dedicated for morality (Decety and Cowell, 2018). The realization of how basic forms of empathy, morality, and justice are substantialized in early ontogeny may provide valid information as to gain new insights into the underlying neurobiological precursors of the social brain, which may enable future translation toward therapeutic and medical interventions.

Likewise, suggestions for future lines of research can be inferred from this review. Foremost, we echo the concerns voiced by other authors (Nelson and Mondloch, 2018) that new studies in the fields of distress- and emotion-perception in others should adopt experimental designs employing dynamic stimuli over static ones, as they more clearly resemble daily interpersonal interactions. Furthermore, research is warranted as to elucidate to what extent empathy in the human social brain is innately reserved toward kin- and self-related interests, as well as how strong is the role played by parental dispositions in the infants’ emergence and maturation of empathic concern. Parallel to the last point, scholars have argued that collaboration and instrumental helping might be grounded on distinct psychological processes (Warneken and Tomasello, 2009a), thus, it would be of great avail to address this matter in conjunction with a neuroscientific standpoint, as to identify the neural underpinnings driving such differences. Additionally, new lines of enquiry should be initiated in order to tackle the issues (maybe even the existence) surrounding the MNS and its relationship to empathy and social cognition. Last but not least, we make a call for further studies resulting in the development of therapeutic interventions for those psychiatric and neurological conditions where perceptual processes underlying empathy and moral cognition have been seen to be affected in an atypical manner.

## Author Contributions

CC and YC conceived and designed the review. CC and RM did the literature collection. CC and YC wrote the first draft of the manuscript. All authors contributed to manuscript write-up.

## Conflict of Interest Statement

The authors declare that the research was conducted in the absence of any commercial or financial relationships that could be construed as a potential conflict of interest.

## References

[B1] Alexandrou A. M. Saarinen T. Makela S. Kujala J. Salmelin R. (2017). The right hemisphere is highlighted in connected natural speech production and perception. *Neuroimage* 152 628–638. 10.1016/j.neuroimage.2017.03.006 28268122

[B2] Atkinson A. P. Vuong Q. C. Smithson H. E. (2012). Modulation of the face- and body-selective visual regions by the motion and emotion of point-light face and body stimuli. *Neuroimage* 59 1700–1712. 10.1016/j.neuroimage.2011.08.073 21924368

[B3] Bachmann J. Munzert J. Kruger B. (2018). Neural underpinnings of the perception of emotional states derived from biological human motion: a review of neuroimaging research. *Front. Psychol.* 9:1763. 10.3389/fpsyg.2018.01763 30298036 PMC6160569

[B4] Barbas H. Saha S. Rempel-Clower N. Ghashghaei T. (2003). Serial pathways from primate prefrontal cortex to autonomic areas may influence emotional expression. *BMC Neurosci.* 4:25. 10.1186/1471-2202-4-25 14536022 PMC270042

[B5] Beauchemin M. Gonzalez-Frankenberger B. Tremblay J. Vannasing P. Martinez-Montes E. Belin P. (2011). Mother and stranger: an electrophysiological study of voice processing in newborns. *Cereb. Cortex* 21 1705–1711. 10.1093/cercor/bhq242 21149849

[B6] Belin P. Fecteau S. Bedard C. (2004). Thinking the voice: neural correlates of voice perception. *Trends Cogn. Sci.* 8 129–135. 10.1016/j.tics.2004.01.008 15301753

[B7] Ben-Ami Bartal I. Decety J. Mason P. (2011). Empathy and pro-social behavior in rats. *Science* 334 1427–1430. 10.1126/science.1210789 22158823 PMC3760221

[B8] Ben-Ami Bartal I. Rodgers D. A. Bernardez Sarria M. S. Decety J. Mason P. (2014). Pro-social behavior in rats is modulated by social experience. *eLife* 3:e01385. 10.7554/eLife.01385 24424411 PMC3884117

[B9] Ben-Ami Bartal I. Shan H. Molasky N. M. Murray T. M. Williams J. Z. Decety J. (2016). Anxiolytic treatment impairs helping behavior in rats. *Front. Psychol.* 7:850. 10.3389/fpsyg.2016.00850 27375528 PMC4896909

[B10] Blake R. Shiffrar M. (2007). Perception of human motion. *Annu. Rev. Psychol.* 58 47–73. 10.1146/annurev.psych.57.102904.190152 16903802

[B11] Blasi A. Mercure E. Lloyd-Fox S. Thomson A. Brammer M. Sauter D. (2011). Early specialization for voice and emotion processing in the infant brain. *Curr. Biol.* 21 1220–1224. 10.1016/j.cub.2011.06.009 21723130

[B12] Boyatzis C. J. Chazan E. Ting C. Z. (1993). Preschool children’s decoding of facial emotions. *J. Genet. Psychol.* 154 375–382. 10.1080/00221325.1993.10532190 8245911

[B13] Brosnan S. F. (2013). Justice- and fairness-related behaviors in nonhuman primates. *Proc. Natl. Acad. Sci. U.S.A.* 110(Suppl. 2), 10416–10423. 10.1073/pnas.1301194110 23754407 PMC3690609

[B14] Carver L. J. Dawson G. Panagiotides H. Meltzoff A. N. McPartland J. Gray J. (2003). Age-related differences in neural correlates of face recognition during the toddler and preschool years. *Dev. Psychobiol.* 42 148–159. 10.1002/dev.10078 12555279 PMC3640993

[B15] Chen C. Chen C. Y. Yang C. Y. Lin C. H. Cheng Y. (2015). Testosterone modulates preattentive sensory processing and involuntary attention switches to emotional voices. *J. Neurophysiol.* 113 1842–1849. 10.1152/jn.00587.2014 25540221

[B16] Chen C. Hu C. H. Cheng Y. (2017a). Mismatch negativity (MMN) stands at the crossroads between explicit and implicit emotional processing. *Hum. Brain Mapp.* 38 140–150. 10.1002/hbm.23349 27534834 PMC7133097

[B17] Chen C. Hung A. Y. Fan Y. T. Tan S. Hong H. Cheng Y. (2017b). Linkage between pain sensitivity and empathic response in adolescents with autism spectrum conditions and conduct disorder symptoms. *Autism Res.* 10 267–275. 10.1002/aur.1653 27305862

[B18] Chen C. Lee Y. H. Cheng Y. (2014). Anterior insular cortex activity to emotional salience of voices in a passive oddball paradigm. *Front. Hum. Neurosci.* 8:743. 10.3389/fnhum.2014.00743 25346670 PMC4193252

[B19] Chen C. Liu C. C. Weng P. Y. Cheng Y. (2016a). Mismatch negativity to threatening voices associated with positive symptoms in schizophrenia. *Front. Hum. Neurosci.* 10:362. 10.3389/fnhum.2016.00362 27471459 PMC4945630

[B20] Chen C. Sung J. Y. Cheng Y. (2016b). Neural dynamics of emotional salience processing in response to voices during the stages of sleep. *Front. Behav. Neurosci.* 10:117. 10.3389/fnbeh.2016.00117 27378870 PMC4906046

[B21] Chen C. Yang C. Y. Cheng Y. (2012). Sensorimotor resonance is an outcome but not a platform to anticipating harm to others. *Soc. Neurosci.* 7 578–590. 10.1080/17470919.2012.686924 22642373

[B22] Cheng Y. Chen C. Decety J. (2014). An EEG/ERP investigation of the development of empathy in early and middle childhood. *Dev. Cogn. Neurosci.* 10 160–169. 10.1016/j.dcn.2014.08.012 25261920 PMC6987874

[B23] Cheng Y. Hung A. Y. Decety J. (2012a). Dissociation between affective sharing and emotion understanding in juvenile psychopaths. *Dev. Psychopathol.* 24 623–636. 10.1017/S095457941200020X 22559135

[B24] Cheng Y. Lee S. Y. Chen H. Y. Wang P. Y. Decety J. (2012b). Voice and emotion processing in the human neonatal brain. *J. Cogn. Neurosci.* 24 1411–1419. 10.1162/jocn_a_00214 22360593

[B25] Cheour M. Alho K. Ceponiene R. Reinikainen K. Sainio K. Pohjavuori M. (1998). Maturation of mismatch negativity in infants. *Int. J. Psychophysiol.* 29 217–226. 10.1016/S0167-8760(98)00017-8 9664229

[B26] Church R. M. (1959). Emotional reactions of rats to the pain of others. *J. Comp. Physiol. Psychol.* 52 132–134. 10.1037/h0043531 13654562

[B27] Cowell J. M. Decety J. (2015a). Precursors to morality in development as a complex interplay between neural, socio environmental, and behavioral facets. *Proc. Natl. Acad. Sci. U.S.A.* 112 12657–12662. 10.1073/pnas.1508832112 26324885 PMC4611595

[B28] Cowell J. M. Decety J. (2015b). The neuroscience of implicit moral evaluation and its relation to generosity in early childhood. *Curr. Biol.* 25 93–97. 10.1016/j.cub.2014.11.002 25532892 PMC4286511

[B29] de Waal F. B. (2008). Putting the altruism back into altruism: the evolution of empathy. *Annu. Rev. Psychol.* 59 279–300. 10.1146/annurev.psych.59.103006.093625 17550343

[B30] Decety J. (2010). The neurodevelopment of empathy in humans. *Dev. Neurosci.* 32 257–267. 10.1159/000317771 20805682 PMC3021497

[B31] Decety J. Bartal I. B. Uzefovsky F. Knafo-Noam A. (2016). Empathy as a driver of prosocial behaviour: highly conserved neurobehavioural mechanisms across species. *Philos. Trans. R. Soc. Lond. B Biol. Sci.* 371:20150077. 10.1098/rstb.2015.0077 26644596 PMC4685523

[B32] Decety J. Cowell J. M. (2014a). Friends or foes: is empathy necessary for moral behavior? *Perspect. Psychol. Sci.* 9 525–537. 10.1177/1745691614545130 25429304 PMC4241340

[B33] Decety J. Cowell J. M. (2014b). The complex relation between morality and empathy. *Trends Cogn. Sci.* 18 337–339. 10.1016/j.tics.2014.04.008 24972506

[B34] Decety J. Cowell J. M. (2018). Interpersonal harm aversion as a necessary foundation for morality: a developmental neuroscience perspective. *Dev. Psychopathol.* 30 153–164. 10.1017/S0954579417000530 28420449

[B35] Decety J. Meidenbauer K. L. Cowell J. M. (2018). The development of cognitive empathy and concern in preschool children: a behavioral neuroscience investigation. *Dev. Sci.* 21:e12570. 10.1111/desc.12570 28523733

[B36] Decety J. Svetlova M. (2012). Putting together phylogenetic and ontogenetic perspectives on empathy. *Dev. Cogn. Neurosci.* 2 1–24. 10.1016/j.dcn.2011.05.003 22682726 PMC6987713

[B37] Decety J. Yoder K. J. (2017). The emerging social neuroscience of justice motivation. *Trends Cogn. Sci.* 21 6–14. 10.1016/j.tics.2016.10.008 27865787

[B38] Dehaene-Lambertz G. Dehaene S. (1994). Speed and cerebral correlates of syllable discrimination in infants. *Nature* 370 292–295. 10.1038/370292a0 8035876

[B39] Dehaene-Lambertz G. Pena M. (2001). Electrophysiological evidence for automatic phonetic processing in neonates. *Neuroreport* 12 3155–3158. 10.1097/00001756-200110080-00034 11568655

[B40] Dinstein I. Thomas C. Behrmann M. Heeger D. J. (2008). A mirror up to nature. *Curr. Biol.* 18 R13–R18. 10.1016/j.cub.2007.11.004 18177704 PMC2517574

[B41] Dondi M. Simion F. Caltran G. (1999). Can newborns discriminate between their own cry and the cry of another newborn infant? *Dev. Psychol.* 35 418–426. 10082012 10.1037//0012-1649.35.2.418

[B42] Downing P. E. Jiang Y. Shuman M. Kanwisher N. (2001). A cortical area selective for visual processing of the human body. *Science* 293 2470–2473. 10.1126/science.1063414 11577239

[B43] Fan Y. T. Chen C. Chen S. C. Decety J. Cheng Y. (2014). Empathic arousal and social understanding in individuals with autism: evidence from fMRI and ERP measurements. *Soc. Cogn. Affect. Neurosci.* 9 1203–1213. 10.1093/scan/nst101 23929944 PMC4127026

[B44] Fan Y. T. Cheng Y. (2014). Atypical mismatch negativity in response to emotional voices in people with autism spectrum conditions. *PLoS One* 9:e102471. 10.1371/journal.pone.0102471 25036143 PMC4103818

[B45] Fan Y. T. Hsu Y. Y. Cheng Y. (2013). Sex matters: n-back modulates emotional mismatch negativity. *Neuroreport* 24 457–463. 10.1097/WNR.0b013e32836169b9 23660632

[B46] Ferguson H. J. Cane J. E. Douchkov M. Wright D. (2015). Empathy predicts false belief reasoning ability: evidence from the N400. *Soc. Cogn. Affect. Neurosci.* 10 848–855. 10.1093/scan/nsu131 25326041 PMC4448031

[B47] Frith C. D. Frith U. (2006). The neural basis of mentalizing. *Neuron* 50 531–534. 10.1016/j.neuron.2006.05.001 16701204

[B48] Hamann K. Warneken F. Greenberg J. R. Tomasello M. (2011). Collaboration encourages equal sharing in children but not in chimpanzees. *Nature* 476 328–331. 10.1038/nature10278 21775985

[B49] Hamlin J. K. (2014). The case for social evaluation in preverbal infants: gazing toward one’s goal drives infants’ preferences for Helpers over Hinderers in the hill paradigm. *Front. Psychol.* 5:1563. 10.3389/fpsyg.2014.01563 25688216 PMC4310275

[B50] Hamlin J. K. (2015). “The infantile origins of our moral brains,” in *The Moral Brain: A Multidisciplinary Perspective*, eds Decety J. Wheatley T. (Cambridge, MA: MIT Press), 105–122.

[B51] Hamlin J. K. Wynn K. Bloom P. (2010). Three-month-olds show a negativity bias in their social evaluations. *Dev. Sci.* 13 923–929. 10.1111/j.1467-7687.2010.00951.x 20977563 PMC2966030

[B52] Hatfield E. Cacioppo J. T. Rapson R. L. (1993). Emotional contagion. *Curr. Dir. Psychol. Sci.* 2 96–99. 10.1111/1467-8721.ep10770953

[B53] Haviland J. M. Lelwica M. (1987). The induced affect response: 10-week-old infants’ responses to three emotion expressions. *Dev. Psychol.* 23 97–104. 10.1037/0012-1649.23.1.97

[B54] Hickok G. (2009). Eight problems for the mirror neuron theory of action understanding in monkeys and humans. *J. Cogn. Neurosci.* 21 1229–1243. 10.1162/jocn.2009.21189 19199415 PMC2773693

[B55] Hoffman M. L. (2007). “The origins of empathic morality in toddlerhood,” in *Socioemotional Development in the Toddler Years: Transitions and Transformations*, eds Brownell C. A. Kopp C. B. (New York, NY: Guilford Press), 132–145.

[B56] Hung A. Y. Ahveninen J. Cheng Y. (2013). Atypical mismatch negativity to distressful voices associated with conduct disorder symptoms. *J. Child Psychol. Psychiatry* 54 1016–1027. 10.1111/jcpp.12076 23701279 PMC3749266

[B57] Hung A. Y. Cheng Y. (2014). Sex differences in preattentive perception of emotional voices and acoustic attributes. *Neuroreport* 25 464–469. 10.1097/WNR.0000000000000115 24488031

[B58] Kanakogi Y. Okumura Y. Inoue Y. Kitazaki M. Itakura S. (2013). Rudimentary sympathy in preverbal infants: preference for others in distress. *PLoS One* 8:e65292. 10.1371/journal.pone.0065292 23776467 PMC3680456

[B59] Kilner J. M. Friston K. J. Frith C. D. (2007). Predictive coding: an account of the mirror neuron system. *Cogn. Process.* 8 159–166. 10.1007/s10339-007-0170-2 17429704 PMC2649419

[B60] Kisilevsky B. S. Hains S. M. Lee K. Xie X. Huang H. Ye H. H. (2003). Effects of experience on fetal voice recognition. *Psychol. Sci.* 14 220–224. 10.1111/1467-9280.02435 12741744

[B61] Kret M. E. Pichon S. Grezes J. de Gelder B. (2011). Similarities and differences in perceiving threat from dynamic faces and bodies. An fMRI study. *Neuroimage* 54 1755–1762. 10.1016/j.neuroimage.2010.08.012 20723605

[B62] Kuhl P. K. (2004). Early language acquisition: cracking the speech code. *Nat. Rev. Neurosci.* 5 831–843. 10.1038/nrn1533 15496861

[B63] Lee Y. E. Yun J. E. Kim E. Y. Song H. J. (2015). The development of infants’ sensitivity to behavioral intentions when inferring others’ social preferences. *PLoS One* 10:e0135588. 10.1371/journal.pone.0135588 26383160 PMC4575196

[B64] Lepage J. F. Theoret H. (2007). The mirror neuron system: grasping others’ actions from birth? *Dev. Sci.* 10 513–523. 10.1111/j.1467-7687.2007.00631.x 17683336

[B65] Li W. Han S. (2010). Perspective taking modulates event-related potentials to perceived pain. *Neurosci. Lett.* 469 328–332. 10.1016/j.neulet.2009.12.021 20026179

[B66] Lloyd-Fox S. Blasi A. Mercure E. Elwell C. E. Johnson M. H. (2012). The emergence of cerebral specialization for the human voice over the first months of life. *Soc. Neurosci.* 7 317–330. 10.1080/17470919.2011.614696 21950945

[B67] Martin G. B. Clark R. D. (1982). Distress crying in neonates: species and peer specificity. *Dev. Psychol.* 18 3–9. 10.1037/0012-1649.18.1.3

[B68] Mella N. Studer J. Gilet A. L. Labouvie-Vief G. (2012). Empathy for pain from adolescence through adulthood: an event-related brain potential study. *Front. Psychol.* 3:501. 10.3389/fpsyg.2012.00501 23189065 PMC3505871

[B69] Meltzoff A. N. (2007). ‘Like me’: a foundation for social cognition. *Dev. Sci.* 10 126–134. 10.1111/j.1467-7687.2007.00574.x 17181710 PMC1852489

[B70] Michalska K. J. Zeffiro T. A. Decety J. (2016). Brain response to viewing others being harmed in children with conduct disorder symptoms. *J. Child Psychol. Psychiatry* 57 510–519. 10.1111/jcpp.12474 26472591 PMC4789171

[B71] Nelson N. L. Mondloch C. J. (2018). Children’s visual attention to emotional expressions varies with stimulus movement. *J. Exp. Child Psychol.* 172 13–24. 10.1016/j.jecp.2018.03.001 29573670

[B72] Nystrom P. (2008). The infant mirror neuron system studied with high density EEG. *Soc. Neurosci.* 3 334–347. 10.1080/17470910701563665 18979389

[B73] Oostenbroek J. Suddendorf T. Nielsen M. Redshaw J. Kennedy-Costantini S. Davis J. (2016). Comprehensive longitudinal study challenges the existence of neonatal imitation in humans. *Curr. Biol.* 26 1334–1338. 10.1016/j.cub.2016.03.047 27161497

[B74] Peelen M. V. Atkinson A. P. Andersson F. Vuilleumier P. (2007). Emotional modulation of body-selective visual areas. *Soc. Cogn. Affect. Neurosci.* 2 274–283. 10.1093/scan/nsm023 18985133 PMC2566760

[B75] Peelen M. V. Atkinson A. P. Vuilleumier P. (2010). Supramodal representations of perceived emotions in the human brain. *J. Neurosci.* 30 10127–10134. 10.1523/JNEUROSCI.2161-10.2010 20668196 PMC6633378

[B76] Pichon S. de Gelder B. Grezes J. (2008). Emotional modulation of visual and motor areas by dynamic body expressions of anger. *Soc. Neurosci.* 3 199–212. 10.1080/17470910701394368 18979376

[B77] Pichon S. de Gelder B. Grezes J. (2009). Two different faces of threat. Comparing the neural systems for recognizing fear and anger in dynamic body expressions. *Neuroimage* 47 1873–1883. 10.1016/j.neuroimage.2009.03.084 19371787

[B78] Price S. A. Brosnan S. F. (2012). To each according to his need? Variability in the responses to inequity in non-human primates. *Soc. Justice Res.* 25 140–169. 10.1007/s11211-012-0153-z

[B79] Sagi A. Hoffman M. L. (1976). Empathic distress in the newborn. *Dev. Psychol.* 12 175–176. 10.1037/0012-1649.12.2.175

[B80] Schirmer A. Striano T. Friederici A. D. (2005). Sex differences in the preattentive processing of vocal emotional expressions. *Neuroreport* 16 635–639. 10.1097/00001756-200504250-00024 15812323

[B81] Schmidt M. F. Sommerville J. A. (2011). Fairness expectations and altruistic sharing in 15-month-old human infants. *PLoS One* 6:e23223. 10.1371/journal.pone.0023223 22003380 PMC3188955

[B82] Scott R. M. Baillargeon R. (2017). Early false-belief understanding. *Trends Cogn. Sci.* 21 237–249. 10.1016/j.tics.2017.01.012 28259555

[B83] Scott R. M. Roby E. (2015). Processing demands impact 3-year-olds’ performance in a spontaneous-response task: new evidence for the processing-load account of early false-belief understanding. *PLoS One* 10:e0142405. 10.1371/journal.pone.0142405 26562840 PMC4642936

[B84] Simner M. L. (1971). Newborn’s response to the cry of another infant. *Dev. Psychol.* 5 136–150. 10.1037/h0031066

[B85] Sinke C. B. Sorger B. Goebel R. de Gelder B. (2010). Tease or threat? Judging social interactions from bodily expressions. *Neuroimage* 49 1717–1727. 10.1016/j.neuroimage.2009.09.065 19804836

[B86] Sloane S. Baillargeon R. Premack D. (2012). Do infants have a sense of fairness? *Psychol. Sci.* 23 196–204. 10.1177/0956797611422072 22258431 PMC3357325

[B87] Sommerville J. A. Schmidt M. F. H. Yun J. E. Burns M. (2013). The development of fairness expectations and prosocial behavior in the second year of life. *Infancy* 18 40–66. 10.1111/j.1532-7078.2012.00129.x

[B88] Tomasello M. Gonzalez-Cabrera I. (2017). The role of ontogeny in the evolution of human cooperation. *Hum. Nat.* 28 274–288. 10.1007/s12110-017-9291-1 28523464 PMC5524848

[B89] Tucker D. M. Luu P. Derryberry D. (2005). Love hurts: the evolution of empathic concern through the encephalization of nociceptive capacity. *Dev. Psychopathol.* 17 699–713. 10.1017/S0954579405050339 16262988

[B90] Vaish A. Carpenter M. Tomasello M. (2010). Young children selectively avoid helping people with harmful intentions. *Child Dev.* 81 1661–1669. 10.1111/j.1467-8624.2010.01500.x 21077854

[B91] Vaish A. Missana M. Tomasello M. (2011). Three-year-old children intervene in third-party moral transgressions. *Br. J. Dev. Psychol.* 29 (Pt 1), 124–130. 10.1348/026151010X532888 21288257

[B92] Warneken F. Grafenhain M. Tomasello M. (2012). Collaborative partner or social tool? New evidence for young children’s understanding of joint intentions in collaborative activities. *Dev. Sci.* 15 54–61. 10.1111/j.1467-7687.2011.01107.x 22251292

[B93] Warneken F. Tomasello M. (2009a). The roots of human altruism. *Br. J. Psychol.* 100 (Pt 3), 455–471. 10.1348/000712608X379061 19063815

[B94] Warneken F. Tomasello M. (2009b). Varieties of altruism in children and chimpanzees. *Trends Cogn. Sci.* 13 397–402. 10.1016/j.tics.2009.06.008 19716750

[B95] Woodward A. L. Gerson S. A. (2014). Mirroring and the development of action understanding. *Philos. Trans. R. Soc. Lond. B Biol. Sci.* 369:20130181. 10.1098/rstb.2013.0181 24778377 PMC4006183

[B96] Zhang D. Liu Y. Hou X. Sun G. Cheng Y. Luo Y. (2014). Discrimination of fearful and angry emotional voices in sleeping human neonates: a study of the mismatch brain responses. *Front. Behav. Neurosci.* 8:422. 10.3389/fnbeh.2014.00422 25538587 PMC4255595

